# Auto- versus human-driven plan in mediastinal Hodgkin lymphoma radiation treatment

**DOI:** 10.1186/s13014-018-1146-3

**Published:** 2018-10-19

**Authors:** Stefania Clemente, Caterina Oliviero, Giuseppe Palma, Vittoria D’Avino, Raffaele Liuzzi, Manuel Conson, Roberto Pacelli, Laura Cella

**Affiliations:** 1 Azienda Ospedaliera Universitaria Federico II, Naples, Italy; 2 National Research Council, Institute of Biostructures and Bioimaging, Naples, Italy; 3 Department of Advanced Biomedical Sciences, Federico II University School of Medicine, Naples, Italy

**Keywords:** Volumetric modulated arc therapy, Automated planning optimization, Hodgkin lymphoma, Normal tissue sparing, NTCP

## Abstract

**Background:**

Technological advances in Hodgkin lymphoma (HL) radiation therapy (RT) by high conformal treatments potentially increase control over organs-at-risk (OARs) dose distribution. However, plan optimization remains a time-consuming task with great operator dependent variability. Purpose of the present study was to devise a fully automated pipeline based on the Pinnacle^3^ Auto-Planning (AP) algorithm for treating female supradiaphragmatic HL (SHL) patients.

**Methods:**

CT-scans of 10 female patients with SHL were considered. A “butterfly” (BF) volumetric modulated arc therapy was optimized using SmartArc module integrated in Pinnacle^3^ v. 9.10 using Collapsed Cone Convolution Superposition algorithm (30 Gy in 20 fractions). Human-driven (Manual-BF) and AP-BF optimization plans were generated. For AP, an optimization objective list of Planning Target Volume (PTV)/OAR clinical goals was first implemented, starting from a subset of 5 patients used for algorithm training. This list was then tested on the remaining 5 patients (validation set). In addition to the BF technique, the AP engine was applied to a 2 coplanar disjointed arc (AP-ARC) technique using the same objective list. For plan evaluation, dose-volume-histograms of PTVs and OARs were extracted; homogeneity and conformity indices (HI and CI), OARs dose-volume metrics and odds for different toxicity endpoints were computed. Non-parametric Friedman and Dunn tests were used to identify significant differences between groups.

**Results:**

A single AP objective list for SHL was obtained. Compared to the manual plan, both AP-plans offer comparable CIs while AP-ARC also achieved comparable HIs. All plans fulfilled the clinical dose criteria set for OARs: both AP solutions performed at least as good as Manual-BF plan. In particular, AP-ARC outperformed AP-BF in terms of heart sparing involving a lower risk of coronary events and radiation-induced lung fibrosis. Hands-on planning time decreased by a factor of 10 using AP on average.

**Conclusions:**

Despite the high interpatient PTV (size and position) variability, it was possible to set a standard SHL AP optimization list with a high level of generalizability. Using the implemented list, the AP module was able to limit OAR doses, producing clinically acceptable plans with stable quality without additional user input. Overall, the AP engine associated to the arc technique represents the best option for SHL.

## Background

Modern radiation therapy (RT) approaches in Hodgkin lymphoma (HL), with lower prescribed doses (20–30 Gy) and smaller irradiated volumes (involved site or involved node), lead to a reduction of organs-at-risk (OARs) exposure [1]. Accordingly, the rates of radiation-induced late toxicity are expected to be lower [2, 3] when compared with older series of successfully treated long term surviving HL patients [4, 5]. In parallel, a considerable effort has been made to identify those HL radiation delivery modalities that increased control over target as well as OAR dose distributions [6–10].

Several, widely available, intensity-modulated radiation therapy (IMRT) planning solutions have been proposed in the literature. Among the different IMRT techniques, the dosimetric advantages of the “butterfly” (BF) technique for female patients with mediastinal HL has been reported [11, 12]. In particular, BF volumetric modulated arc therapy (VMAT) showed high levels of conformation permitting to achieve the most balanced compromise between higher conformation around the target and OAR sparing [11].

Dose-volume histogram (DVH) predictors and Normal Tissue Complication Probability (NTCP) models developed for HL patient population have supported the planning optimization procedures intended to limit OAR complications. NTCP models have been reported for late side effects such as radiation induced lung damage [13–15], hypothyroidism [16, 17], and cardiovascular diseases [2, 18–20].

However, plan optimization remains a very time-consuming and operator dependent task. This issue has been addressed by the recent introduction of automated engines in treatment planning (TP) systems in order to create an optimized plan with minimal user interaction. They have proved able to generate IMRT plans of non-inferior or even higher clinical quality compared to human driven plans for many different tumour sites, such as head and neck [21], prostate [22, 23]and lung [24]. To the best of our knowledge, however, no study investigated the Auto-Planning (AP) algorithm applied to VMAT for supradiaphragmatic HL (SHL) patients.

Given this background, the current study was designed to devise a fully automated pipeline, based on the Pinnacle^3^ (Philips Radiation Oncology Systems, Fitchburg, WI, USA) AP algorithm, for treating female SHL patients. For 10 female patients AP plans were compared with treatment plans generated by experienced human planners. The different TP solutions were evaluated by quantitative risk estimates based on published models for different toxicity endpoints.

## Methods

### Patient data

Planning CT-scans of 10 consecutive female patients with SHL (Table 1) in standard supine position were extracted from our clinical database. Involved site clinical target volume (CTV) was defined according to ILROG guidelines [1] for early stage HL. Planning Target Volume (PTV) was obtained by CTV uniform 10-mm expansion. Target and OARs structures were contoured on free-breathing CT images (voxel size = 0.94 × 0.94 × 5 mm^3^). The following OARs were contoured: lungs, heart, left ventricle, left anterior descending (LAD) artery, esophagus, spinal cord, breasts and thyroid. Heart and its substructures were contoured according to heart contouring guidelines [25]. All contours were reviewed and approved by one of the authors (M.C.).

**Table 1:** Nodal disease localization and Planning Target Volume (PTV) size for each patient

Patient	Nodal disease localization	PTV size (cc)
Training		
1	VI level, upper mediastinal	252.0
2	supraclavicular right, upper mediastinal	503.2
3	supraclavicular left and right, III level right, upper mediastinal	350.7
4	supraclavicular left and right, upper mediastinal, diaphragmatic	480.3
5	supraclavicular right, upper mediastinal	247.6
Validation		
6	supraclavicular left, III level left, upper mediastinal	496.8
7	supraclavicular left, upper mediastinal	559.5
8	supraclavicular left and right, III level right, upper mediastinal	423.0
9	II and III level left, supraclavicular left, upper mediastinal	199.3
10	supraclavicular left, upper mediastinal	309.6

A total dose of 30 Gy was prescribed in 1.5 Gy daily fractions for all patients.

### Treatment plan optimization

Each patient was purposely planned with an antero-posterior/postero-anterior weighted BF 6 MV photon beams VMAT technique by Pinnacle^3^ v. 9.10. SmartArc module and Collapsed Cone Convolution Superposition dose calculation algorithm (grid resolution 3 mm) were used. The BF VMAT technique consists of 3 arcs: 2 coplanar arcs, one anterior and one posterior (width ranging from 60° to 100°) and one anterior no-coplanar (couch angle 90°) arc (width ranging from 45° to 60°). Arc width was customized to provide tumour coverage according to patient anatomy. All plans were optimized for a Varian True Beam STx Linac (Varian Medical System, Palo Alto, CA) equipped with a High Definition 120 multileaf collimator (HD120MLC).

For each patient, two different optimization approaches were used: the human-driven optimization (Manual-BF) and the AP optimization (AP-BF), both generated using the same required clinical constraints (Table 2). No constraints were used on the left ventricle and LAD artery.

**Table 2:** Planning Target Volume (PTV) and Organ-At-Risk dose-volume constraints for plan optimization and patients violating the required constraints when the Auto Plan best optimization objective list was applied

Structure	Parameter	Required Objective	Patient violating the requirements		
			AP-BF Training	AP-BF Validation	AP-ARC Validation
PTV					
	D_mean_ (Gy)	30	–	–	–
	V_95%_ (%)	95	–	–	–
	V_107%_ (%)	1	3,4	6,7,8	6,7
Breast					
	V_4Gy_ (%)	50	–	–	–
	V_10Gy_ (%)	33	–	–	–
Lung-PTV					
	V_5Gy_ (%)	50	–	–	–
	V_10Gy_ (%)	33	–	–	–
Thyroid-PTV					
	D_mean_ (Gy)	20	3	–	–
	V_18Gy_ (%)	50	3	–	–
	V_25Gy_ (%)	33	–	–	–
Heart-PTV					
	D_mean_ (Gy)	3	2	6,8	6,8
	V_7.7Gy_(%)	50	–	–	–
	V_15Gy_(%)	33	–	–	–
Spinal Cord					
	D_max_ (Gy)	30	–	–	–
Esophagus-PTV					
	D_max_ (Gy)	32	–	–	–

The Manual-BF plan was generated using planner-dependent definitions of additional guidance contours (inner and outer rings structures for PTV), avoidance structures and associated optimization objectives. The plan was validated by 2 experienced clinical physicists (S.C., C.O.) in consensus.

The AP-BF plan was optimized using Pinnacle^3^ AP algorithm. In summary, it is a fully integrated module in the TP system which uses a progressive optimization algorithm to continually adjust the optimization objective list set by the user to meet or further decrease OARs doses and related DVH parameters with minimal compromise to PTV coverage, thus simulating the decision-making process of an experienced human planner [21]. Indeed, the AP algorithm iteratively fine-tunes the target coverage and OAR sparing by creating multiple additional structures based both on the relative geometry of originally segmented ROIs and on transient dose distributions. The algorithm automatically assigns dose-volume objectives to the additional ROIs which are added to the standard optimization list [26].

In addition to the BF technique, the AP engine was applied to a 2 coplanar disjointed arcs (AP-ARC) technique which consists of 2 full co-planar arcs moving clockwise and counter clockwise respectively avoiding the arms.

In the present study, each AP plan was obtained running a single optimization cycle.

### AP optimization objective list

The starting point of Pinnacle^3^ AP optimization procedure is setting a user dependent optimization list of PTV/OAR clinical goals. In order to set a single AP-BF list for SHL with a high level of generalizability, we selected 5 out of 10 patients to be used as a training set. The patient selection criterion was based on nodal disease localization and target size heterogeneity (Table 1). The remaining 5 patients were instead used as a validation set to test the obtained optimization list. In Table 1, PTV characteristics for training and validation patient sets are reported.

**Fig. 1:**
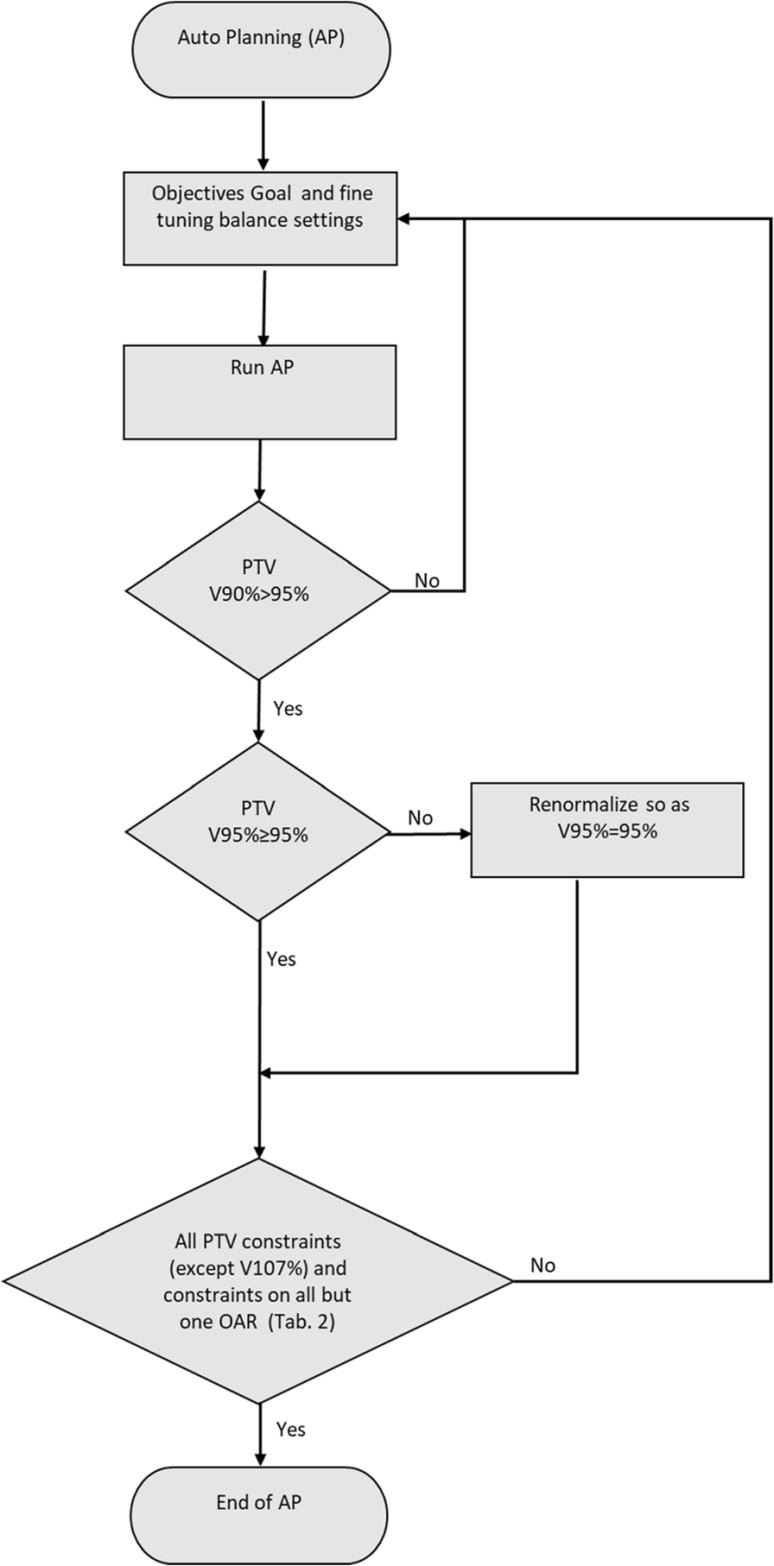
The flow of the algorithm used for setting the Auto Planning optimization objective list (learning phase)

For all training set patients, the list was iteratively refined using the algorithm described in Fig. 1 (learning phase). The algorithm was designed to satisfy, first, the tumour-coverage criteria (at least 95% of PTV received at least 95% of prescription dose) and, secondly, the constraints on the OARs of Table 2. To this end, in the algorithm we introduced the concept of “admitted violations”, intended as the maximum number of required objectives not satisfied at the end of an optimization cycle. The admitted violations for the algorithm were PTV V_107%_ > 1% and only one OAR not fulfilling the required dose-volume constrains reported in Table 2.

The list thus obtained was then tested on the validation set for both AP-BF and AP-ARC configurations.

### Plan analysis

For plan comparison, DVHs of PTVs and OARs were extracted. For each patient, relevant PTV/OAR DVH metrics were analyzed: the percentage volume receiving at least X dose (Vx), near maximum dose (D_2%_), near minimum dose (D_98%_), mean (D_mean_) and median dose (D_50%_).

The target coverage was assessed via the conformity index (CI=V_95%_/PTV_vol_) and the homogeneity index (HI = [D_2%_-D_98%_]/ D_50%_).

Toxicity risks were calculated according to several NTCP models available in the literature [2, 3, 13, 16, 18–20, 27, 28]. NTCP models specifically extrapolated from HL patients’ cohorts were used.

In addition, the number of planned monitor units (MU) and the hands-on planning time were recorded. The hands-on planning time was defined as the time of human interaction with the TP system.

The median and the range were employed to describe all continuous variables and the non-parametric ANOVA (Friedman matched-pairs signed-rank test) was used to determine statistically significant differences (*p* < 0.05). A posthoc procedure was performed in order to identify significant differences between groups (Dunn’s test).

## Results

### AP optimization objective list

**Fig. 2:**
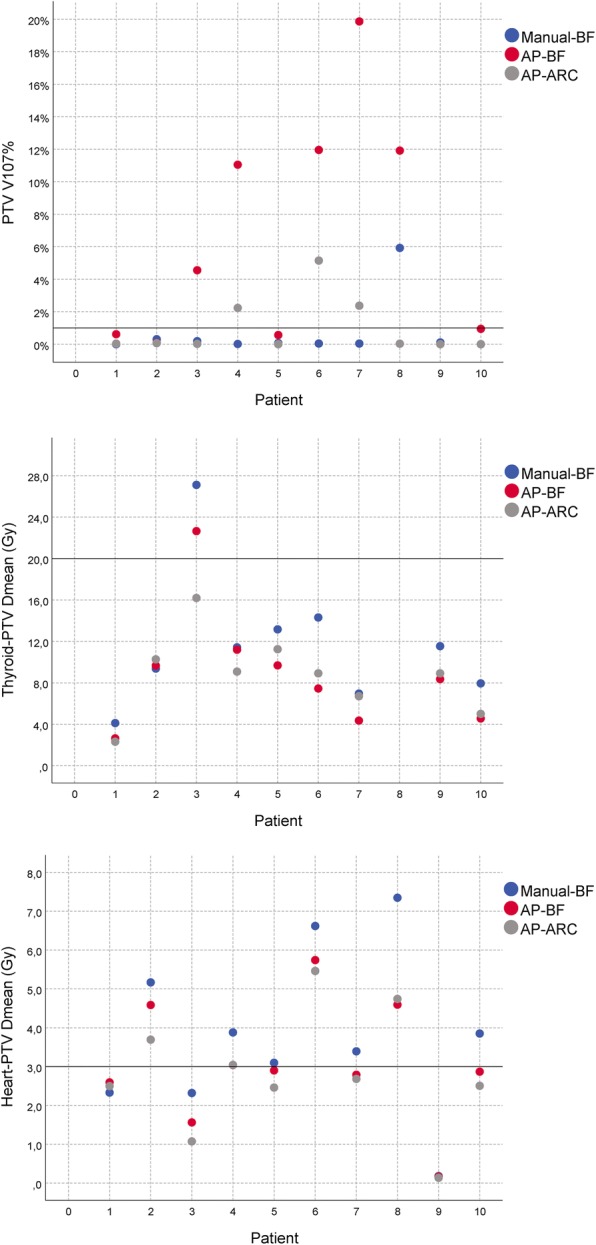
Comparison of Planning Target Volume (PTV) percentage volume receiving at least 107% of the prescribed dose (V_107%_), of Thyroid-PTV mean dose (D_mean)_ and Heart-PTV mean dose (D_mean_) values for Manual-BF, AP-BF, AP-ARC

At the end of the learning phase, we succeeded in implementing a single AP optimization objective list for SHL patients (details in Table 3). This list was subsequently applied, with no further refinement, to the validation set. Each AP plan of the validation set (patients 6–10) fulfilled all PTV/OAR constraints within the admitted violations of the required constraints, i.e. all PTV constraints (except V_107%_) and constraints on all but one OAR were fulfilled (Table 2 and Fig. 2).

**Table 3:** Auto Planning setting list

Auto-planning Settings					
Max Itaration	60				
Engine Type	Biological				
Tuning Balance	5%				
Dose Fall-Off Margin	1.8 cm				
Hot-Spot Maximum Goal	102%				
Use Cold-Spot ROIs	YES				
*Target Optimization Goals*					
PTV	30 Gy				
CTV	30 Gy				
*OARs Optimization Goals*					
ROI	Type	Dose (Gy)	Volume (%)	Priority	Compromise
Heart	Mean dose	3		Medium	Yes
Heart	Max DVH	7.7	50	Medium	Yes
Heart	Max DVH	15	33	Medium	Yes
Left Lung	Mean dose	5		Medium	Yes
Left Lung	Max DVH	5	15	Medium	Yes
Left Lung	Max DVH	10	12	Medium	Yes
Left Lung	Max DVH	20	10	Medium	Yes
Right Lung	Mean dose	5		Medium	Yes
Right Lung	Max DVH	5	15	Medium	Yes
Right Lung	Max DVH	10	12	Medium	Yes
Right Lung	Max DVH	20	10	Medium	Yes
Esophagus	Max DVH	28	50	Medium	Yes
Esophagus	Max DVH	30	50	Medium	Yes
Thyroid	Mean dose	20		Medium	Yes
Thyroid	Max DVH	18	50	Medium	Yes
Thyroid	Max DVH	25	33	Medium	Yes
Left Breast	Mean dose	0.3		High	No
Left Breast	Max DVH	2	4	High	No
Left Breast	Max DVH	5	2	High	No
Right Breast	Mean dose	0.3		High	No
Right Breast	Max DVH	2	4	High	No
Right Breast	Max DVH	5	2	High	No
Spinal Cord	Max dose	25		High	Yes
PRV Spinal Cord	Max dose	26		High	Yes
Ring PTV (+ 1.5 cm)	Max dose	15		High	Yes
Body-PTV-Ring PTV	Max dose	10		High	Yes

*Abbreviations*: *ROI* Region of Interest, *PTV* planning Target Volume, *CTV* Clinical Target Volume, *OARs* Organs At Risk, *DVH* Dose Volume Histogram

### Target volume

**Fig. 3:**
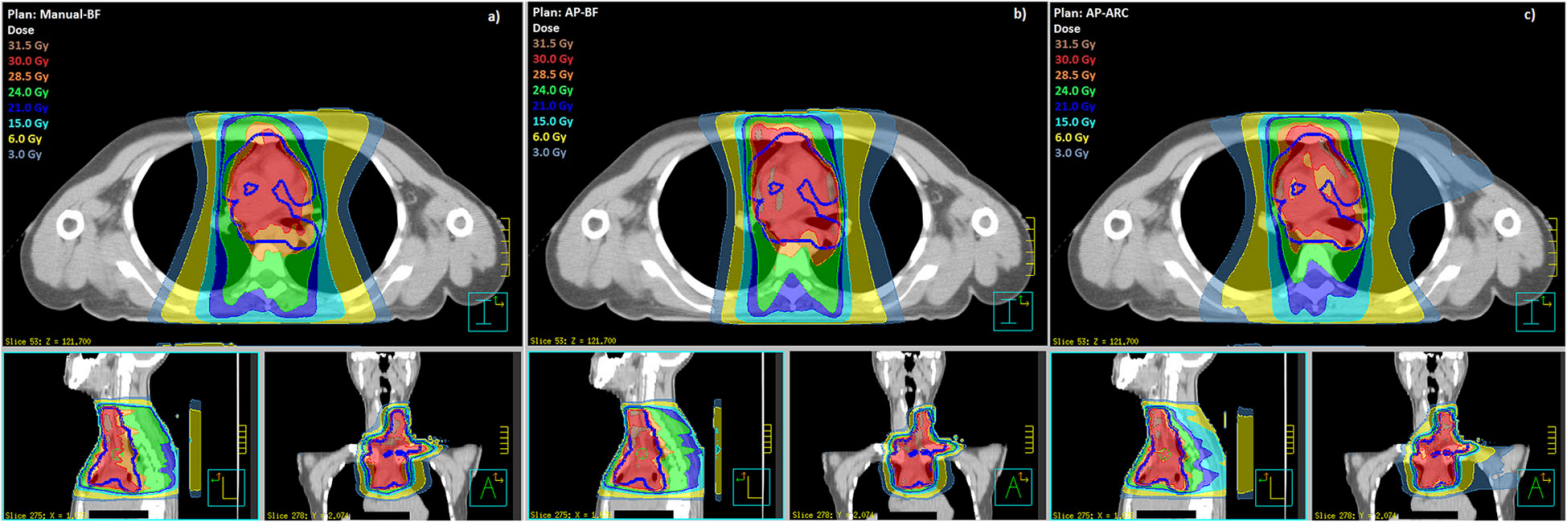
Dose distributions in one representative patient for the three treatment plans: **a**) Manual-BF, **b**) AP-BF, **c**) AP-ARC

Median target size was 386.9 cc (199.3–559.5 cc). Figure 3 illustrates dose distributions in one representative patient for the three treatment techniques.

**Fig. 4:**
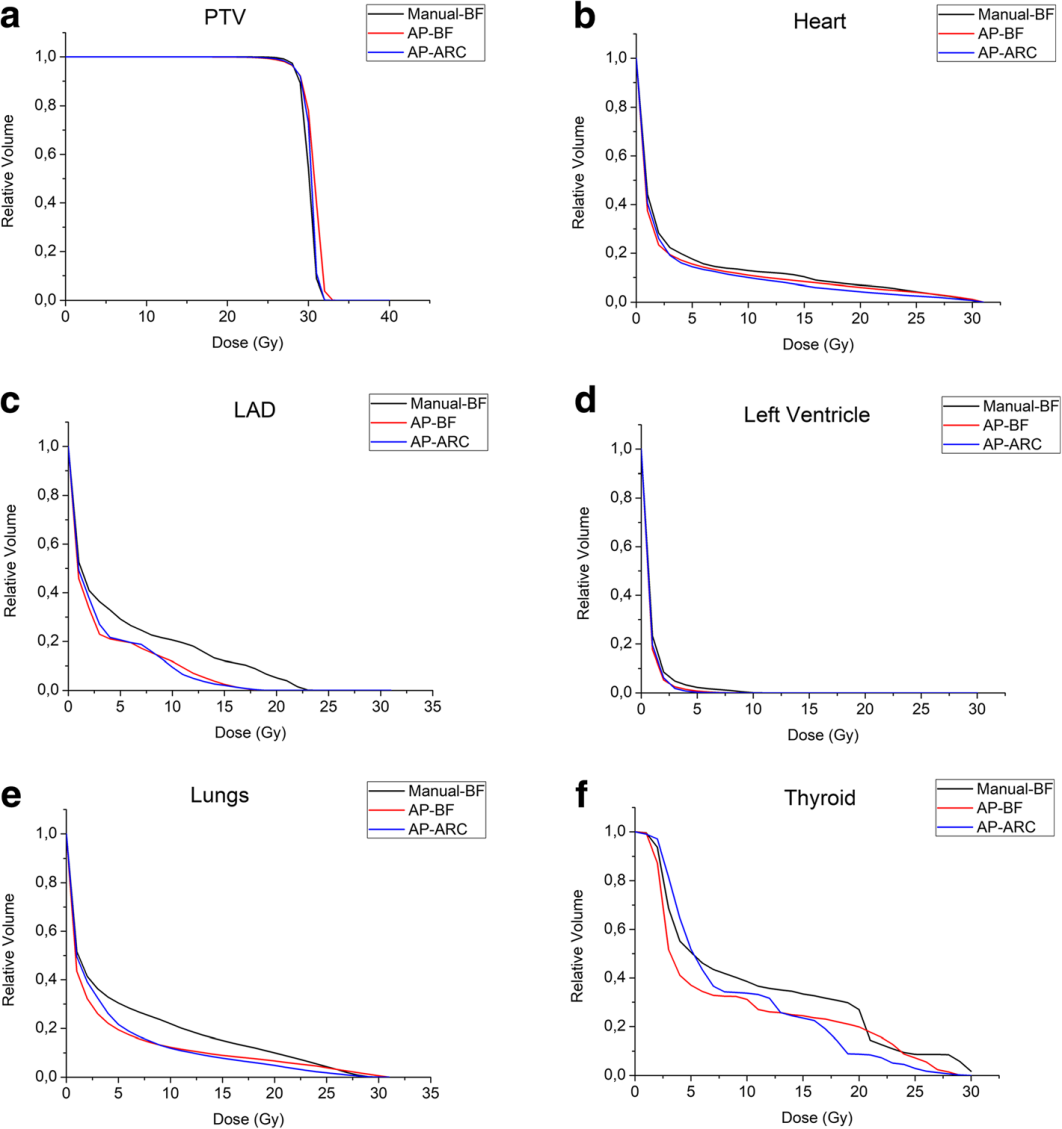
Median cumulative patient dose-volume histograms (DVHs) for the Planning Target Volume-PTV (A) and the organs-at-risk (B–F) for the three treatment plans: Manual-BF, AP-BF, AP-ARC

Median PTV DVH from the 3 plans were largely overlapping (Fig. 4.A). AP offers comparable coverage of the PTV with the manual plan. CI indices for AP plans were comparable to that of the Manual-BF plan, while AP-BF showed a higher HI compared to both Manual-BF and AP-ARC (Table 4).

**Table 4:** Dosimetric indices and comparative analysis for Planning Target Volume (PTV) and different organs at risk for manual and automated plans

Structure	Dose Index	Median (range)			*p*-value< 0.05^a^	
		Manual-BF	Pinnacle^3^Auto-Planning			
		1	AP-BF	AP-ARC	1vs.2,3	2vs.3
			2	3		
PTV	HI	0.12 (0.09–0.16)	0.16 (0.1–0.22)	0.13 (0.09–0.18)	1 < 2	2 > 3
	CI	0.95 (0.94–0.96)	0.95 (0.94–0.95)	0.95 (0.95–0.97)	–	–
Heart	D_mean_ (Gy)	3.8 (0.2–8.7)	3.3 (0.2–7.2)	3 (0.1–6.3)	1 > 2,3	2 > 3
	D_2%_ (Gy)	28.4 (0.4–31.5)	28.3 (0.4–31.3)	26.8 (0.3–30.2)	1 > 3	2 > 3
Left Ventricle	D_mean_ (Gy)	1 (0.1–3.1)	0.7 (0.1–1.6)	0.8 (0.1–1.7)	1 > 2,3	–
LAD	D_50%_ (Gy)	1.3 (0.0–25.7)	0.8 (0.0–11.9)	1 (0.0–13.7)	1 > 2	–
Lungs	D_mean_ (Gy)	6.3 (1.6–9.2)	4.5 (1.4–5.9)	4.6 (1.4–6.0)	1 > 2,3	–
	V_5Gy_ (%)	32.0 (7.5–52.4)	20.9 (6.3–29.0)	23.7 (7.7–35.0)	1 > 2	2 < 3
	V_20Gy_ (%)	11.6 (2.6–18.5)	8.4 (2.0–12.3)	7.2 (1.0–9.7)	1 > 3	2 > 3
Thyroid	D_mean_ (Gy)	16.8 (11.9–28.4)	15.4 (10.1–25.7)	15.5 (9.6–21.2)	1 > 2,3	–
	V_30Gy_ (%)	19.6 (7.9–51.3)	19.2 (8.7–27.9)	14.0 (3.7–22.5)	–	–
Breast	D_mean_ (Gy)	1.1 (0.1–2.3)	0.4 (0.1–1.2)	0.6 (0.1–1.6)	1 > 2,3	2 < 3
Normal Tissue	D_mean_ (Gy)	2.9 (1.5–5.4)	2.4 (1.4–3.8)	2.4 (1.2–3.7)	1 > 2	

*Abbreviations: PTV* planning Target Volume, *HI* Homogeneity Index, Conformity Index, *D*_*mean*_ mean dose, *D*_*2%*_ near maximum dose, *D*_*50%*_ median dose, *V*_*X*_ percentage volume exceeding X, *LAD* Left Anterior Descending artery

^a^Friedman and Dunn tests

### Organs at risk

All AP plans fulfilled the clinical dose criteria set for OARs within the admitted violation. Data in Table 4 show that the AP solutions were never outperformed by the manual plans and the AP engine also leads to a general reduction of OARs dose metrics. In particular, AP-ARC was never outperformed by AP-BF, except for lungs V_5Gy_ and breast D_mean_, which show a slightly higher sparing provided by AP-BF.

In terms of NTCP, AP engine was always at least as safe as manual planning (Table 5), with the exception of radiation-induced lung fibrosis where AP-BF involved a higher risk compared with manual plan. In particular, comparing AP-ARC and AP-BF, AP –ARC resulted in a lower risk of radiation-induced coronary events and lung fibrosis compared to AP-BF.

**Table 5:** Risk analysis for different organs and endpoints for manual and automated plans

Structure	Clinical endpoint	Reference	Median (range)			*p*-value< 0.05^d^	
			Manual-BF	Pinnacle^3^Auto-Planning			
			1	AP-BF	AP-ARC	1vs.2, 3	2vs.3
				2	3		
			NTCP (%)				
Heart	Valvular defects	Cella et al. (2013)	26.71 (0.96–82.63)	21.14 (0.96–84.21)	16.09 (0.94–81.36)	–	–
Lungs	Radiation fibrosis^a^	Cella et al. (2015)^b^	5.85 (2.72–8.39)	6.41 (2.19–8.03)	5.53 (1.30–7.51)	1 < 2	2 > 3
		Cella et al. (2015)^c^	6.05 (3.33–16.79)	3.88 (3.21–7.87)	4.80 (3.22–7.94)	1 > 2,3	2 < 3
Thyroid	Hypothyroidism	Cella et al. (2012)	3.22 (2.08–9.98)	3.16 (2.15–4.36)	2.62 (1.78–3.58)	–	–
			RRR				
Heart	Major coronary events	van Nimwegen et al. (2015)	1.28 (1.01–1.64)	1.24 (1.01–1.53)	1.22 (1.01–1.47)	1 > 2,3	2 > 3
			OR				
LAD	Coronary stenosis	Moignier et al. (2015)	1.06 (1.00–3.43)	1.04 (1.00–1.77)	1.05 (1.00–1.93)	1 > 2	

*Abbreviations: LAD* Left Anterior Descending artery, *NTCP* Normal Tissue Complication Probability, *RRR* Relative Risk Ratio, *OR* Odds Ratio

^a^Computed assuming an age of 30 years at time of irradiation; ^b^Model including lungs D_2%_; ^c^Model including left lung V_5Gy_; ^d^Friedman and Dunn test

The median number of MU were 287.7 (239.6–378.9) for Manual-BF, 267.7 (214.9–382.5) for AP-BF and 375.6 (339.9–456.7) for AP-ARC (*p* < 0.001; AP-ARC > Manual-BF and AP-BF).

Hands-on planning time by AP decreased by an order of magnitude. The mean computation time (performed on a Server Expert hardware platform 32 GB RAM –http://incenter.medical.philips.com/doclib/getdoc.aspx?func=ll&objid=10925579&objaction=open) for the automated procedures was 25 min (AP-BF) and 40 min (AP-ARC).

## Discussion

The most up-to-date and optimized RT techniques applied to mediastinal HL have demonstrated a significant dose reduction to various sensitive critical structures [10–12]. Modern TP systems automate many beam parameters, in particular the beam modulation, via inverse planning computations which create IMRT or VMAT plans so that each treatment plan will result highly customized for each patient. However, the mediastinum remains a critical and complicated target area in HL, due to the heterogeneity of tumour volumes and their position relative to many different important OARs, such as the heart and its substructure or the lungs. As a consequence, HL planning optimization entails a high level of complexity with a wide variation in plan quality that strongly depends on planner skills, as demonstrated for other disease sites by [29]. This issue calls for an additional level of automation in HL RT in order to reduce the inter-operator variability of plan quality. In recent years, different automated treatment planning approaches have been proposed and are commercially available. They show that it is possible to almost fully automate and accelerate this task, improving speed, consistency and quality of RT plans [30].

One proposed knowledge-based solution relies on the concepts of machine learning and uses a library of historical plans for a given disease site to build a model that can predict achievable DVHs for new patients and guide plan optimization [31]. Another approach instead is based on a multicriteria optimization algorithm which provides a database of Pareto-optimal plans [32]. Pinnacle^3^ AP algorithm uses an iterative approach of progressive optimization without requiring any prior database of successful plans [26].

In this study we devised a fully automated pipeline for treating female SHL patients using Pinnacle^3^ AP. First, we designed a learning phase based on a trial-and-error approach to fit an optimization list that could satisfy a number of dosimetric acceptability criteria on the training set patients (as illustrated in the flowchart of Fig. 1). Then, we applied the obtained optimization list as an input for the AP algorithm on an independent validation set of patients.

On the whole, the analysis of the results on the validation set confirmed the behaviours observed in the training phase (Table 2 and Fig. 2). Namely, AP techniques seemed to favour sparing of healthy tissues over target coverage, in agreement with [26]. In particular, the requirement on PTV V_107%_ ≤ 1% was violated when AP was applied to patients 6, 7 and 8 in the validation set. Indeed, in several patients a plan renormalization was necessary to fulfil the requirement of PTV V_95%_ = 95% at the expense of the target high dose region.

Nonetheless, with AP-ARC technique the V_107%_ was always no more than 5% while higher V_107%_ values (≥ 12%) were obtained with the AP-BF technique. In this regard, AP-ARC proved able to largely reduce the gap between the manually optimized and AP-BF plans, as also reflected by the HIs (Table 4). On the other hand, AP plans naturally succeeded in satisfying OAR requirements, with the exception of the heart mean dose for one training and two validation patients, and thyroid D_mean_ and V_18Gy_ for one training patient. However, even in those cases AP engine was able to outperform manual optimization.

**Fig. 5:**
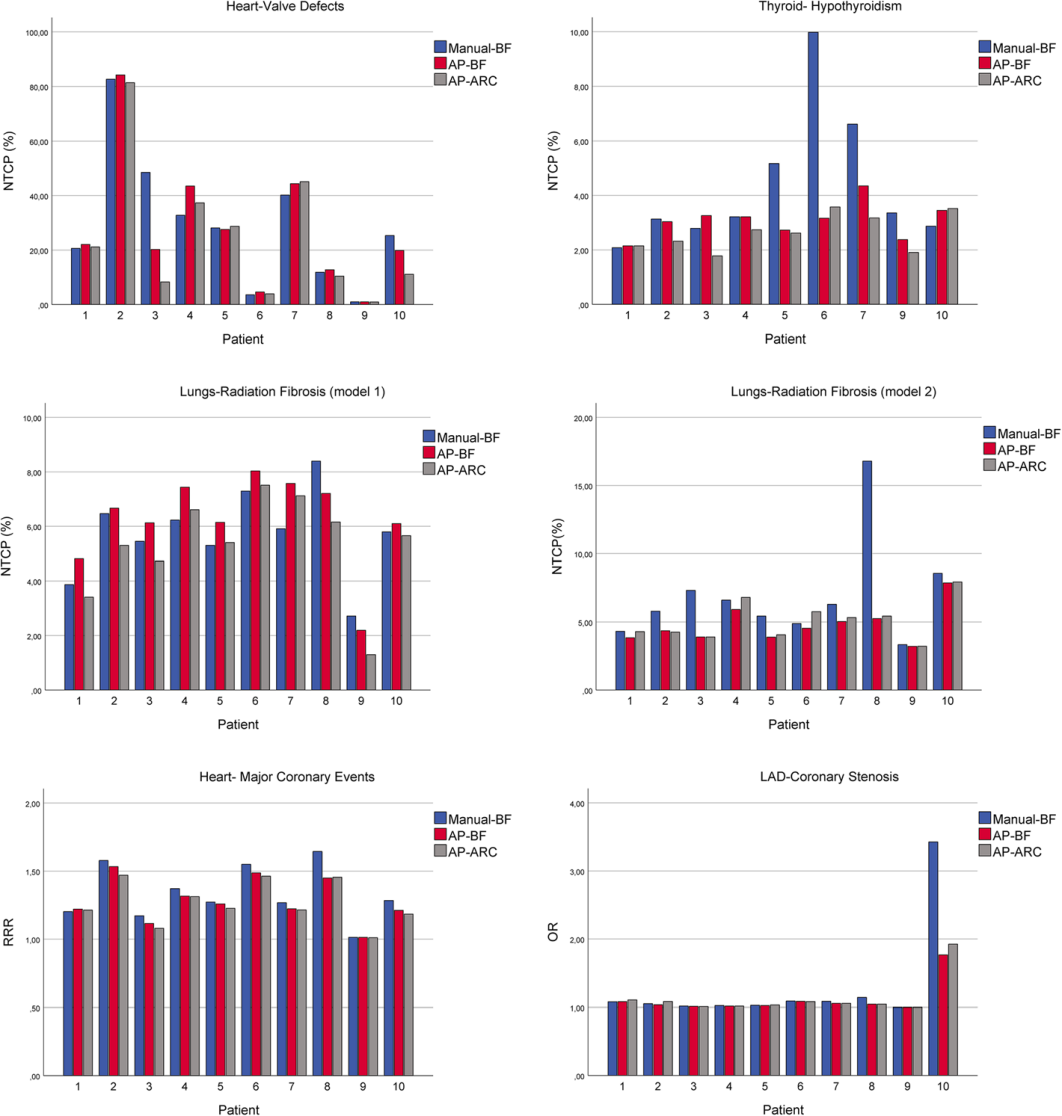
Comparison of morbidity risk parameters for heart, lungs and thyroid for Manual-BF, AP-BF and AP-ARC (please note that patient number 8 underwent a thyroidectomy)

The quantitative assessment of DVH (Fig. 4 and Table 4) revealed that, as a general rule, AP schemes performed at least as well as the manual approach. For lungs and heart, the dosimetric advantages translated into a significant reduction of morbidity risk estimates (Fig.5 and Table 5). In addition, even when non statistically significant differences were found, the observed trends held for all the evaluated variables.

We have to remark that further refinements of the AP plans could be expected by running more than one AP optimization cycle.

Analogously, a general trend suggested that AP-ARC outperforms AP-BF, with the only exception of lungs V_5Gy_ and breast D_mean_ which shows a slightly higher sparing provided by AP-BF. The out of phase behavior of the two considered lungs metrics (namely V_5Gy_ and V_20Gy_) translates into a similar result for estimating the radiation fibrosis risk and reflects the famous conundrum “a lot to a little or a little to a lot” inherent to lungs radiobiology.

The better performances of AP applied to the arc beam settings compared to the well-established “butterfly” technique can be explained by the increased number of beam entries resulting in an augmented number of degrees of freedom that the optimization algorithm can exploit to satisfy the objective list. This point is best demonstrated by the higher homogeneity of the target coverage and by the lower heart doses. In addition, the longer beam-on time for AP-ARC plans (by a factor of about 1.5) is overbalanced by reduced in room times compared to AP-BF plans, which involve a non-coplanar beam. This potentially reduces the immobilization errors and facilitates more comfortable treatments. Of note, no difference between AP-ARC and AP-BF was observed in the non-target tissue mean doses.

Besides, the adoption of the AP algorithm obviously leads to a huge decrease of the hands-on time on the TP system which can be easily quantified in terms of an order of magnitude.

Summing up, the above results prove that we have succeeded in defining a procedure that leads to a fully automation of the TP process for obtaining clinically acceptable SHL plans, despite the high inter-patient target variability (size and position) inherent to the considered disease. The standardization of the treatment is a direct consequence of the automation, thus guaranteeing the quality of treatment delivered in an arbitrary institution independently from the planner’s skills.

Finally, the flowchart devised for setting a single optimization objective list is not tied to the considered disease and, as such, can be applied to any tumour site in order to remove the only operator dependent task left by the Pinnacle^3^ AP optimization tool.

## Conclusions

In this study, we demonstrated the feasibility of a completely automated pipeline based on Pinnacle^3^ AP for SHL plan optimization. The AP module was able to limit OAR doses, thus producing clinically acceptable plans of high quality without additional user interaction. On the whole, the AP engine associated to the arc technique represented the best option for SHL.

## Abbreviations

AP: Auto-Planning
BF: Butterfly
CI: Conformity index
CT: Computed Tomography
CTV: Clinical target volume
DVH: Dose-volume histogram
HI: Homogeneity index
HL: Hodgkin lymphoma
IMRT: Intensity-modulated radiation therapy
LAD: Left anterior descending
MU: Monitor units
NTCP: Normal Tissue Complication Probability
OAR: Organs-at-risk
PTV: Planning Target Volume
RT: Radiation therapy
SHL: Supradiaphragmatic Hodgkin lymphoma
TP: Treatment planning
VMAT: Volumetric modulated arc therapy
